# Teaching Gentle Canine and Feline Handling as a Veterinary Clinical Skill

**DOI:** 10.15694/mep.2021.000158.1

**Published:** 2021-06-07

**Authors:** Jennifer T. Johnson, Julie Hunt, Jamie Perkins, Ashley N. Nibbe

**Affiliations:** 1 Lincoln Memorial University; 2 University of Arizona; 3 The Animal Clinic

**Keywords:** gentle animal handling, fear free, low stress, clinical skills, veterinary education, simulation, models

## Abstract

This article was migrated. The article was marked as recommended.

Gentle animal handling techniques decrease the fear, anxiety, and stress (FAS) felt by companion animals during veterinary visits. These techniques, relatively new in the veterinary field, can be taught to veterinary students in a progressive clinical skills curriculum using models and live animals. This article includes a series of comprehensive lesson plans that are simple to adopt and easy to modify to fit the needs of individual institutions teaching these skills. These laboratories, each reviewing and building on content previously presented, are meant to be accompanied with specific feedback offered by instructors overseeing student performance of skills. Students’ deliberate practice of these techniques is meant to build, refine, and reinforce gentle animal handling from the start of their veterinary education to prepare them to handle animals using these techniques during their clinical training and beyond.

## Introduction

The veterinary profession has recently developed gentler animal handling techniques with the aim of decreasing patients’ life-long FAS associated with veterinary visits and procedures ( Yin, 2009; Rodan *et al.,* 2011; Lloyd, 2017). However, the uptake and implementation of these techniques across the profession has been slow and incomplete. Thus, young people shadowing veterinarians prior to attending veterinary school are likely to have observed and tacitly accepted a variety of animal handling techniques. Due to the variety of handling techniques observed and because most veterinary students will handle companion animals during their career ( *AVMA,* 2020), teaching safe, effective handling skills that do not result in excessive patient FAS during veterinary school is crucial.

Learning and retaining clinical skills requires deliberate practice with specific feedback ( Ericsson, Krampe and Tesch-Römer, 1993; Ericsson, 2004; Duvivier *et al.,* 2011). Clinical skills can be practiced on live animals, cadavers, or models ( Hart, Wood and Weng, 2005; Knight, 2007). Models in particular can be convenient for initial skills training, as they decrease the need for live animals and reduce student anxiety while allowing instructors to focus on teaching and feedback ( Langebaek *et al.,* 2012). When model-trained students subsequently move to performing a task on live animals, they are more skilled, which increases patient and student safety, and decreases animal FAS ( Salas *et al.,* 2005; Langebaek *et al.,* 2012). Other authors have outlined progressive teaching plans for veterinary clinical skills including medical and surgical tasks ( Smeak, 2007; Gopinath *et al.,* 2012; Read, 2013). The goal of this article is to outline a method of progressively educating veterinary students in gentle animal handling in a clinical skills curriculum. The inclusion of comprehensive teaching plans that are simple to adopt and modify (Appendix 1) make this curriculum easy to fit to the needs of diverse institutions.

## Methods

Lincoln Memorial University College of Veterinary Medicine (LMU CVM) has designed a comprehensive clinical skills curriculum as a way for students to progressively gain hands-on experience. The clinical skills curriculum consists of six semesters of mandatory courses that students must pass to progress through the veterinary curriculum. Clinical skills laboratory (CSL) sessions are taught by veterinarians and, when appropriate for the skill, veterinary technicians. Prior to teaching in the CSL, instructors attend a two-hour workshop emphasizing and practicing the delivery of quality feedback. Instructors then reach consensus on how to teach the skills through a series of meetings and workshops that allow discussion and practice of skills ( Johnson and Williamson, 2018).

During each CSL session students practice skills under supervision and are given targeted advice according to the principles of effective feedback ( Nicol and Macfarlane-Dick, 2006; Cornell, 2017). At the end of the session, students undergo formative assessment on their ability to perform a selected skill. Each student is given feedback about what was performed well, where there was room for improvement, and is assigned a global rating score using a six-point scale (1=very poor, 2=poor, 3=borderline unsatisfactory, 4=borderline satisfactory, 5=good, 6=excellent). Points received from these assessments are combined with online or in-laboratory knowledge-based quizzes and objective structured clinical examinations (OSCEs) to generate a student’s course grade. Students are strongly encouraged to practice their skills outside of scheduled laboratory sessions, and they are provided with a well-stocked practice laboratory space and models to take home to facilitate self-directed practice.

Students are required to review pre-laboratory materials such as videos or reading before attending most CSL sessions. Students are also required to complete the online Fear Free ^®^ certification course prior to handling companion animals in the first semester of their veterinary program. Fear Free is one of two gentle animal handling programs; Low Stress Handling ^®^ is the other similar certification. LMU CVM students subsequently practice gentle animal handling through a series of progressive CSL sessions in semesters 1-6; these sessions use a combination of models and live dogs and cats ( Table 1). All laboratory procedures are approved by LMU’s Institutional Animal Care and Use Committee.

**Table 1.** LMU CVM clinical skills laboratories with gentle animal handling content

**Semester**	**Lab title**	**Species**	**Live Animal usage**	**Model usage**
1	Canine Handling	Canine	X	X
	Feline Handling	Feline		X
	Introduction to Physical Examination	Canine	X	X
2	Physical Examination - Focus on ophthalmology and oral cavity	Canine	X	
	Venipuncture	Canine Feline	X	X X
3	Physical Examination - Focus on ophthalmology and otoscopy	Canine	X	X
	Canine Clicker Training	Canine	X	
4	Communication Skills and the Physical Examination	Canine	X	
5	Canine Gentle Handling	Canine	X	
	Surgery work up - examination and diagnostics	Canine Feline	X X	
6	Feline Gentle Handling	Feline	X	
	Physical Examination - Focus on ophthalmology with fundic exam and otoscopy	Canine	X	X
	Surgery work up - examination and diagnostics	Canine Feline	X X	

Each CSL session incorporating gentle animal handling is described in Appendix 1. For each session, we have listed the estimated time to complete all the activities including teaching and assessment, suggested an appropriate instructor to student ratio that facilitates adequate feedback to supervise student safety and provide feedback, defined laboratory objectives, and described the order of events in the session and what students will do to meet the objectives. A comprehensive equipment list has been provided to assist with laboratory planning and implementation, along with a suggested assessment activity and in some cases, variations to the laboratory that may prove helpful. These elements make up a complete laboratory plan that can be utilized at various institutions looking to teach gentle animal handling skills.

## Results

The total time that students spend in the 12 animal handling laboratories described is 28 hours, though other topics are also covered in these laboratories, including physical examination, injection administration, venipuncture, and client communication. Students devote additional hours outside of the laboratory to becoming Fear Freecertified, preparing for laboratory sessions using the provided materials, and practicing their skills prior to the OSCEs. These gentle animal handling laboratories have been delivered during veterinary students’ pre-clinical curriculum since the college admitted its first veterinary class in August 2014. For the first three cohorts of veterinary students, the classes of 2018 through 2020, the clinical skills courses were graded on a pass/fail basis, after which time the courses became letter graded. The change in scoring was made because after three years of collecting reliability data on the assessment instruments, the faculty were comfortable making the change to a letter-graded course, and course directors recognized that students would be more motivated to perform in the course if they were rewarded with letter grades for their effort rather than a simple pass/fail marking system.

The initial rollout of the gentle animal handling curriculum did not contain the Semester 6 Feline Gentle Handling laboratory that utilizes live cats for student skills practice. Prior to the addition of this laboratory, the curriculum used only model cats due to the potentially fractious nature of live cats, and students’ first live cat experience occurred when they encountered shelter cats that required a pre-surgical workup. Students often felt rushed to get their blood samples to the laboratory for analysis, and to get their pre-surgical workup finished within the 2-hour time allotted for patient evaluation, and the shelter cats often became fractious and required sedation. The addition of the Semester 6 Feline Gentle Handling laboratory allowed students to practice gentle handling techniques on live cats that were chosen for their cooperative nature and were premedicated with gabapentin, so that students could practice gentle handling techniques and positioning the cats for mock procedures such as venipuncture. Students subsequently went to their surgical patients with a better understanding of how to handle cats without contributing to their fractious behavior. Similarly, the development of the feline medial saphenous venipuncture model several years into the curriculum allowed students to practice this lower-stress method of drawing blood from cats and better prepared students to interact with live cats in their pre-surgical workup laboratories (Williamson *et al.,* 2019).

LMU CVM has delivered gentle animal handling laboratory sessions to approximately 120 students in each veterinary class, with classes divided into two groups (60 students) for most laboratory sections, unless the laboratory content has required otherwise, such as for the Semester 6 Feline Gentle Handling laboratory. Live cats are more comfortable when spaced farther apart so that they do not see and upset each other, so the class is split into quarters (30 students) for this laboratory.

## Discussion

The progressive series of clinical skills laboratories described represent a spiral curriculum, where skills are taught and expanded upon over time to reinforce previous content and integrate new information and skills ( Harden and Stamper, 1999). Most veterinary students have an interest in pursuing companion animal medicine as a career ( *AVMA,* 2020), so they are intrinsically motivated to learn these techniques. Students are also motivated by the OSCE they must pass once a semester in order to progress through the curriculum. This multi-station multi-rater examination of students’ clinical skills ( Harden *et al.,* 1975) frequently includes handling and physical examination stations that require students to incorporate gentle animal handling techniques.

Delivery of these clinical skills sessions has not been without its challenges. We understood while designing the program that obtaining consensus among the educators and clinicians would be critical. If we taught students to use gentle handling techniques as part of the formal curriculum, only for them to observe clinicians performing conflicting methods later as part of the informal or hidden curriculum, students would soon learn that veterinarians treat animals differently than they have been taught. Studies suggest that when there is a pedagogical mismatch between the formal and informal curricula, students internalize the information provided by the informal curriculum ( Shaw, 2006; Wear and Skillicorn, 2009). To prevent this from occurring, we first had to reach consensus among our own faculty on what methods we would teach and use in our facility. Keeping in mind that the clinicians in our facility were originally trained using traditional handling methods and not the newer gentle handling methods, we went through a consensus building process. After a building a consensus and reaching the conclusion to teach and use gentle handling methods, we have hosted annually a two to four hour workshop for faculty to practice gentle techniques on dogs and cats, to ask questions, to discuss available training and to develop a consensus for teaching and use ( Johnson and Williamson, 2018). When possible, the session is led by a Fear Freecertified Diplomat of the American College of Veterinary Behaviorists (DACVB).

Similarly, most students begin veterinary school thinking that they already know how to handle companion animals correctly. Helping students to realize that most of them are not yet skilled at reading canine and feline body language and interpreting how they should proceed for the best possible outcome can be a challenge. Students with more handling experience can be particularly difficult to convince to adopt a new and different method. However, even adults with previous canine experience have been shown to miss stress signals and misread facial cues ( Mariti *et al.,* 2012; Bloom and Friedmann, 2013). Faculty presenting a united message about the importance and effectiveness of these techniques, about their adoption of these techniques at this institution, and their expectations for performance on assessments, is critical to convincing students to learn new methods.

Another challenge has been having our students learn with shelter animals that have a variety of levels of FAS, and therefore student experiences are inconsistent. However, shelter patients are used for veterinary instruction at numerous other schools ( Smeak, 2007, 2008; Freeman *et al.,* 2013; Stevens and Gruen, 2014; Shively *et al.,* 2018), and handling a variety of patients is similar to what students may encounter in a veterinary practice. Some of the dogs we worked with in the clinical skills laboratory are not fearful, anxious, or stressed and only need treats as a reward for calm behavior, especially younger dogs. Some students have struggled to recognize this, and dogs may be given treats inappropriately and inadvertently taught to chase hands. Because of this some mild gastrointestinal upset has occurred in over-rewarded dogs as students are encouraged to learn to increase treat value based on levels of FAS and patient reaction to procedures rather than merely giving many treats at once.

By practicing gentle animal handling starting in the first semester of their clinical skills curriculum, LMU CVM students gain handling knowledge, learn the value of positive reinforcement, and have multiple opportunities to practice these skills and receive valuable feedback, fundamental tenets of deliberate practice ( Ericsson, 2004). The progression of the clinical skills laboratories allows students to make stepwise additions to their handling skills, preparing them for their live surgery laboratories and the multiple private practice settings they will experience during their distributed clinical year. Building, refining, and reinforcing gentle animal handling skills from the start of students’ veterinary education helps to prepare them to gently handle animals for countless future encounters.

LMU CVM has been both bolstered and limited by having a relatively new program. There has been less opportunity to gather data and make changes to the existing curriculum, but there is increased openness to adjustments. As the program continues, data about the effectiveness of the gentle animal handling curriculum can be gathered, including pre- and post-training skills assessments. Another limitation is that our faculty does not include a DACVB or Certified Applied Animal Behavioralist (CAAB), though we have had a Fear Free certified DACVB visit and offer feedback on our program, as well as assist with teaching laboratories when available. Based on her visits, modifications have been made to skills including towel wrapping, walking onto scales, positive reinforcement, and muzzle choices. Other growth opportunities for the program include the development of a canine jugular venipuncture model and the collection of outcomes measurements such as how many students maintain their Fear Freecertification after graduation.

## Take Home Messages

• Adoption of newer gentle animal handling techniques in the veterinary medical profession has been slow and inconsistent. Young people observing veterinarians prior to attending veterinary school have likely observed and tacitly accepted various animal handling techniques. Therefore, teaching safe, effective animal handling skills that do not result in excessive patient fear, anxiety, and stress during veterinary school is crucial.
• Clinical skills, such as gentle animal handling skills, are learned through deliberate practice with specific feedback. Handling techniques can be learned and practiced on models before students proceed to practice on live animals.
• Obtaining consensus among both educators and clinicians at the educational institution is critical to ensuring that students do not observe clinicians performing conflicting animal handling methods later in their veterinary school education. This creates a hidden curriculum that suggests to students that gentle animal handling can be abandoned according to need or desire.
• Allowing students to practice gentle animal handling skills in a series of clinical skills laboratories, each with new and more challenging content, represents a spiral curriculum, where skills are taught and expanded upon over time to reinforce previous content and integrate new information and skills.

## Notes On Contributors

Jennifer T. Johnson is Adjunct Professor in Veterinary Medicine at Lincoln Memorial University College of Veterinary Medicine and is Associate Veterinarian at Wellington Veterinary Hospital in Wellington, Colorado. She is Silver Certified in Low Stress animal handling and is Fear Free certified.

Julie Hunt is Associate Dean of Clinical Sciences and Associate Professor of Veterinary Medicine at Lincoln Memorial University College of Veterinary Medicine. She is certified in Fear Free gentle animal handling. ORCID ID: https://orcid.org/0000-0003-1927-432X

Jamie Perkins is Assistant Professor of Veterinary Medicine at University of Arizona College of Veterinary Medicine.

Ashley N. Nibbe is a Licensed Veterinary Technician at The Animal Clinic in Hendersonville, Tennessee. She is certified in Fear Free gentle animal handling.

## Figures and Tables

**Figure 1.**
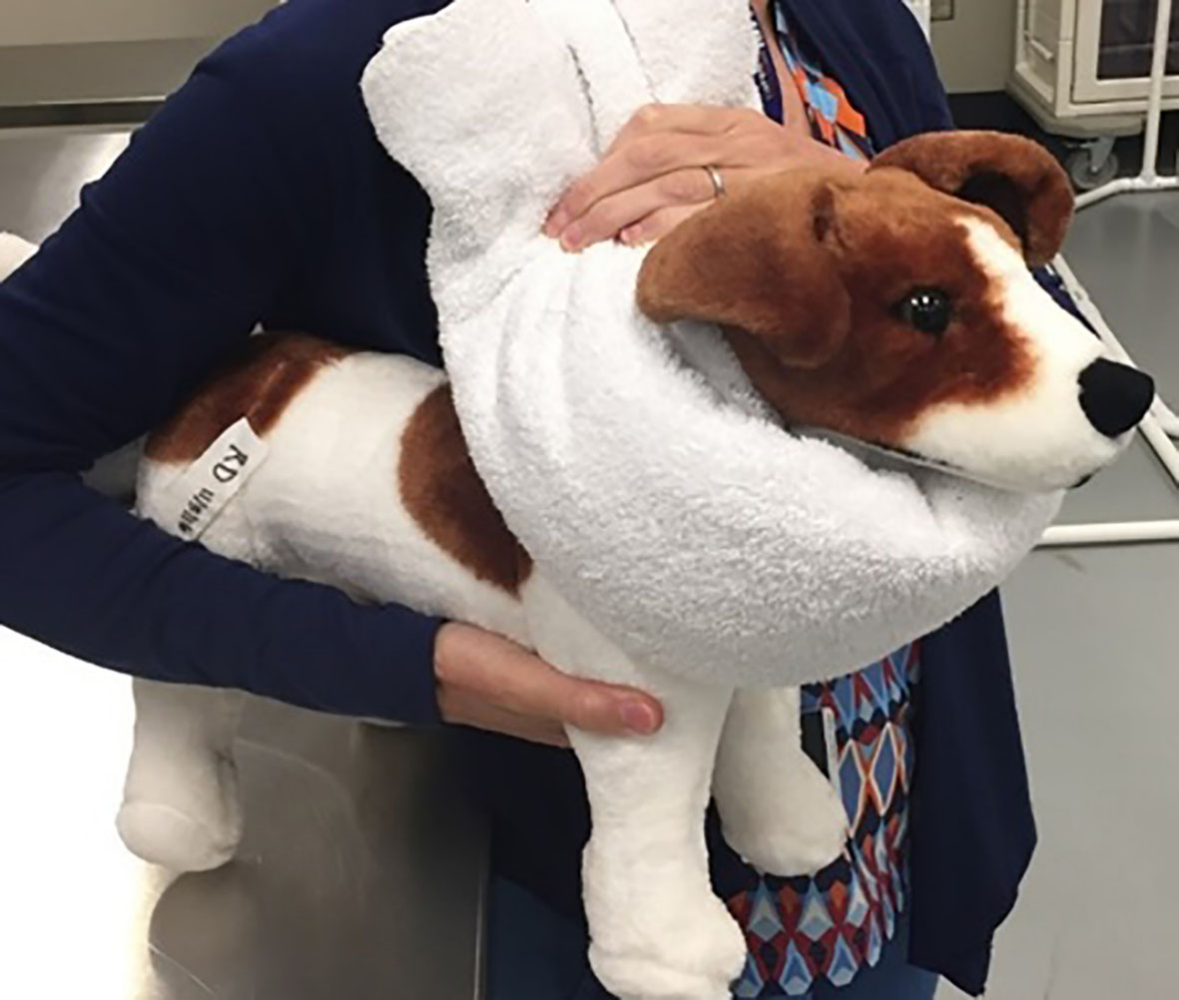
A towel is wrapped around the neck of a small dog model as a restraint

**Figure 2.**
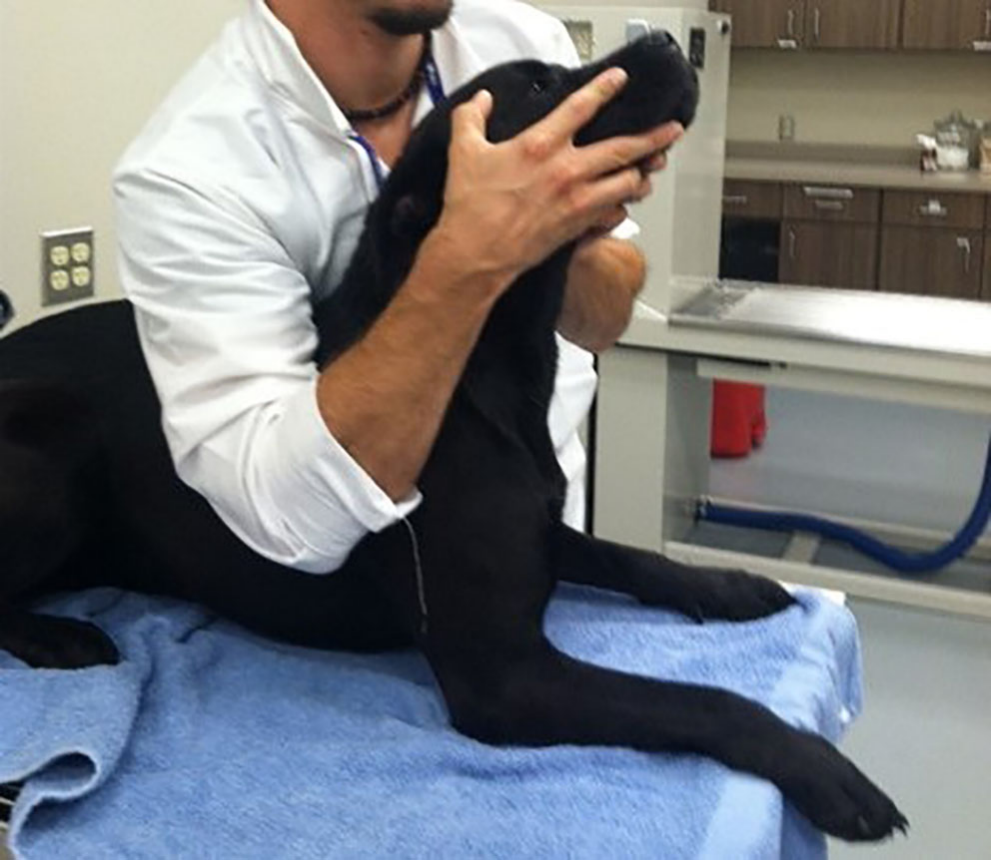
A dog is positioned for jugular venipuncture

**Figure 3.**
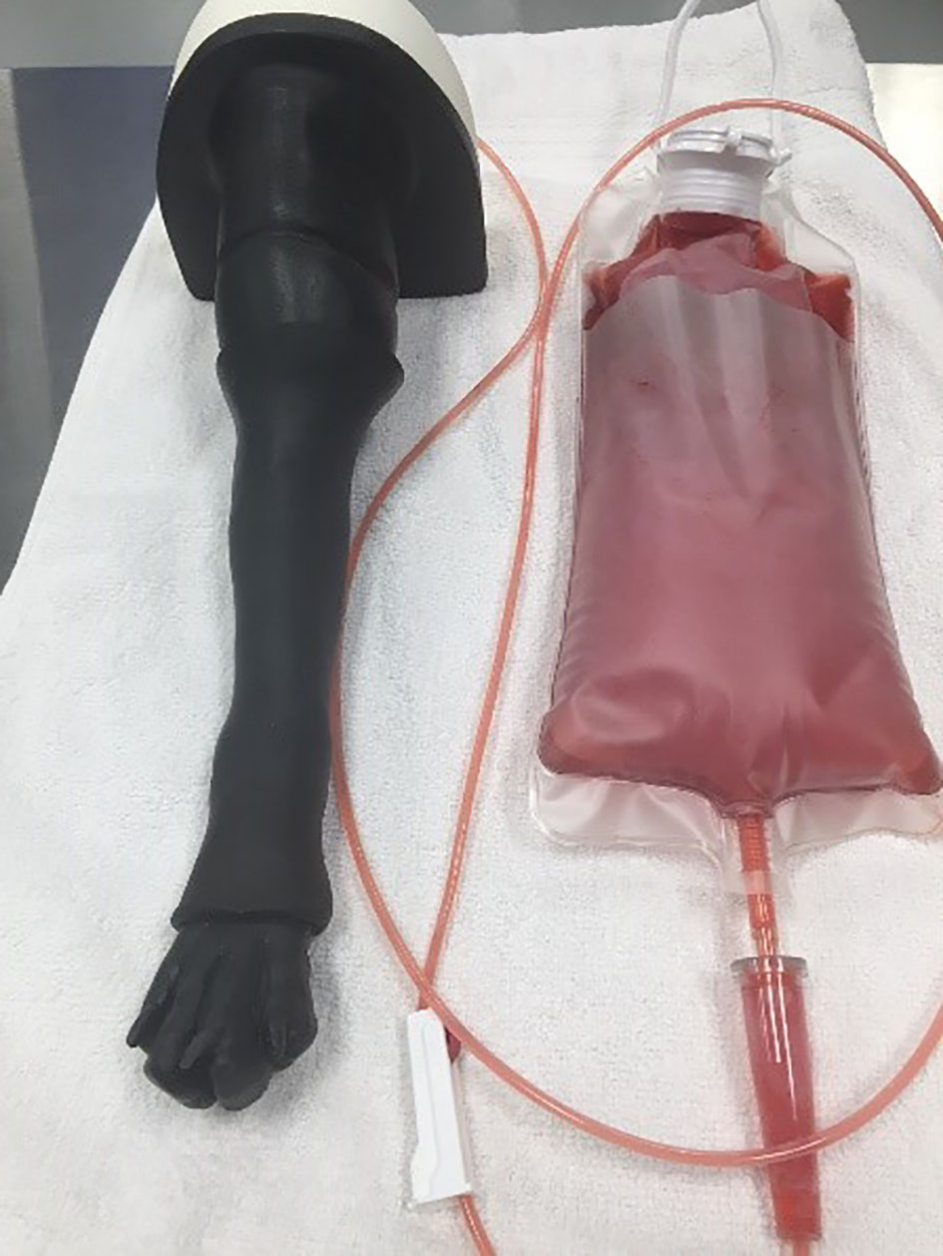
The canine cephalic venipuncture model, available from Surgireal

**Figure 4.**
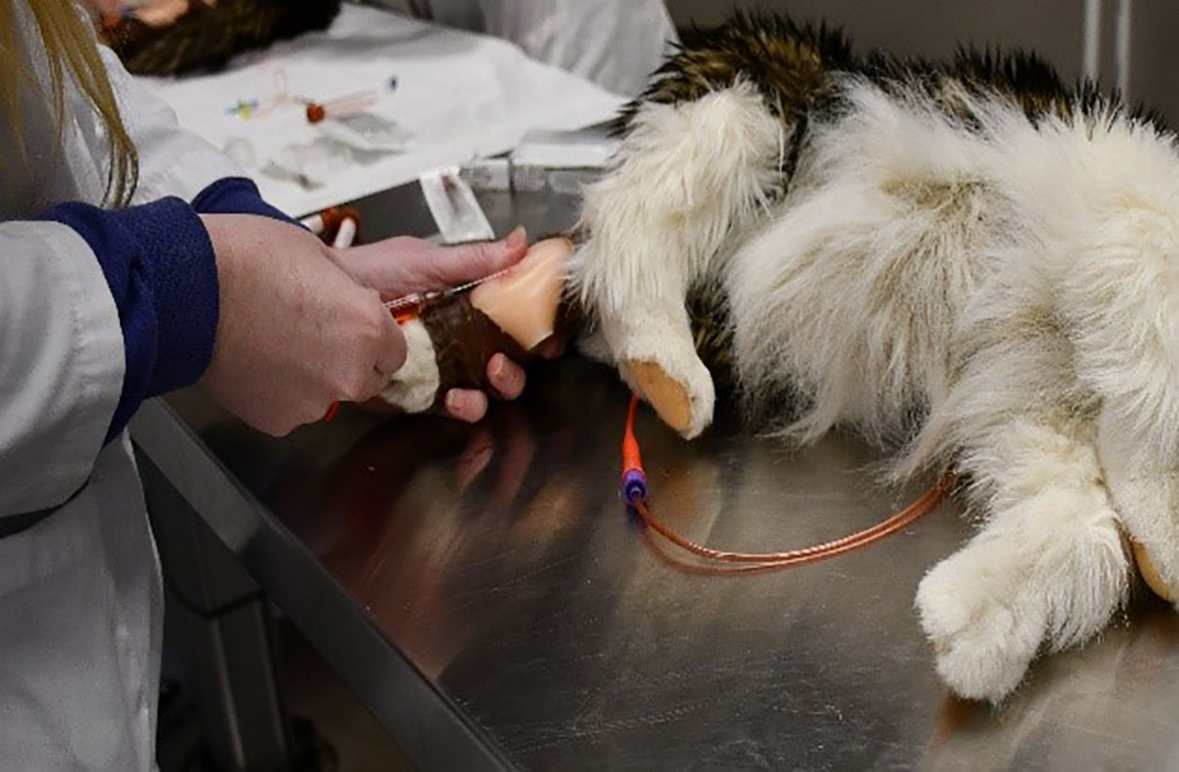
The feline medial saphenous venipuncture model, constructed at Lincoln Memorial University College of Veterinary Medicine (LMU-CVM)

**Figure 5.**
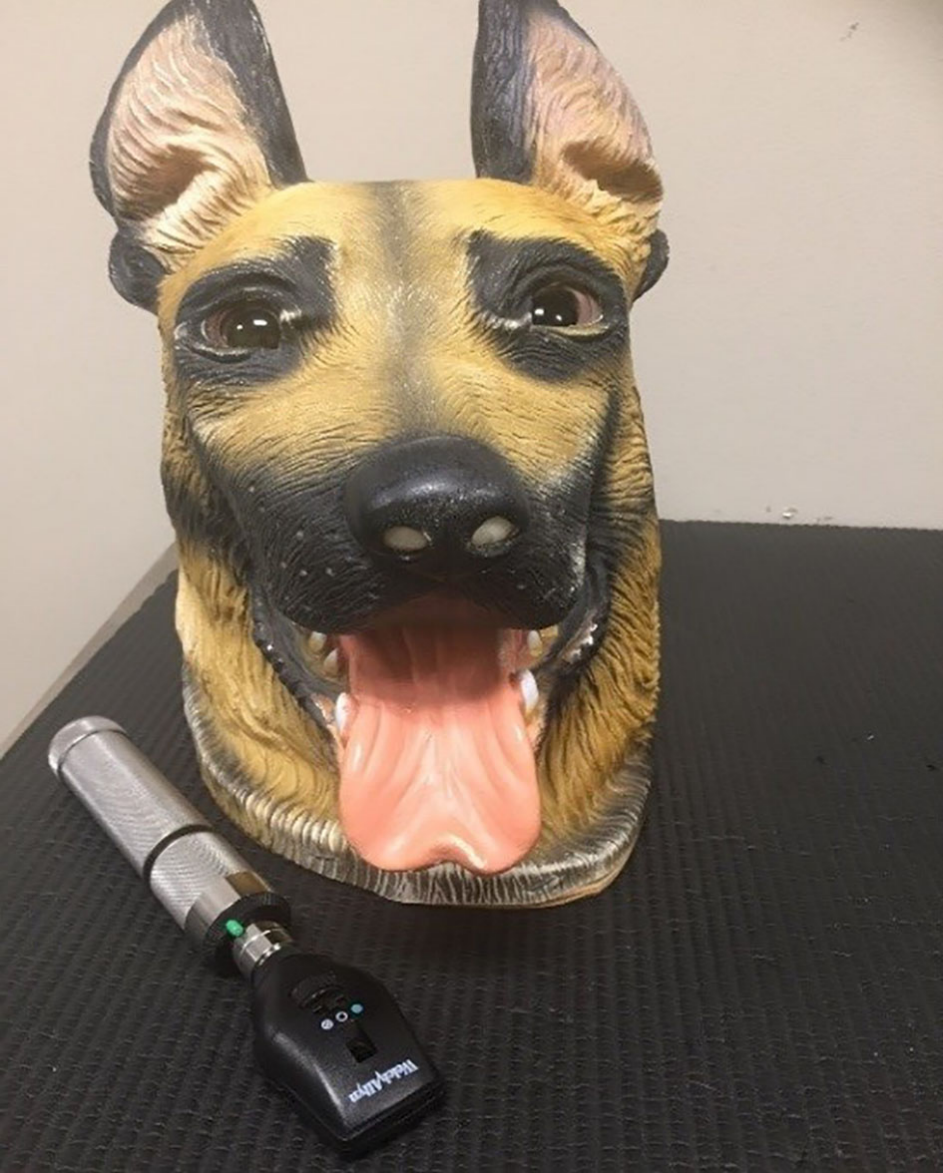
The canine direct and indirect ophthalmoscopy model with interchangeable fundus, created at LMU-CVM

**Figure 6.**
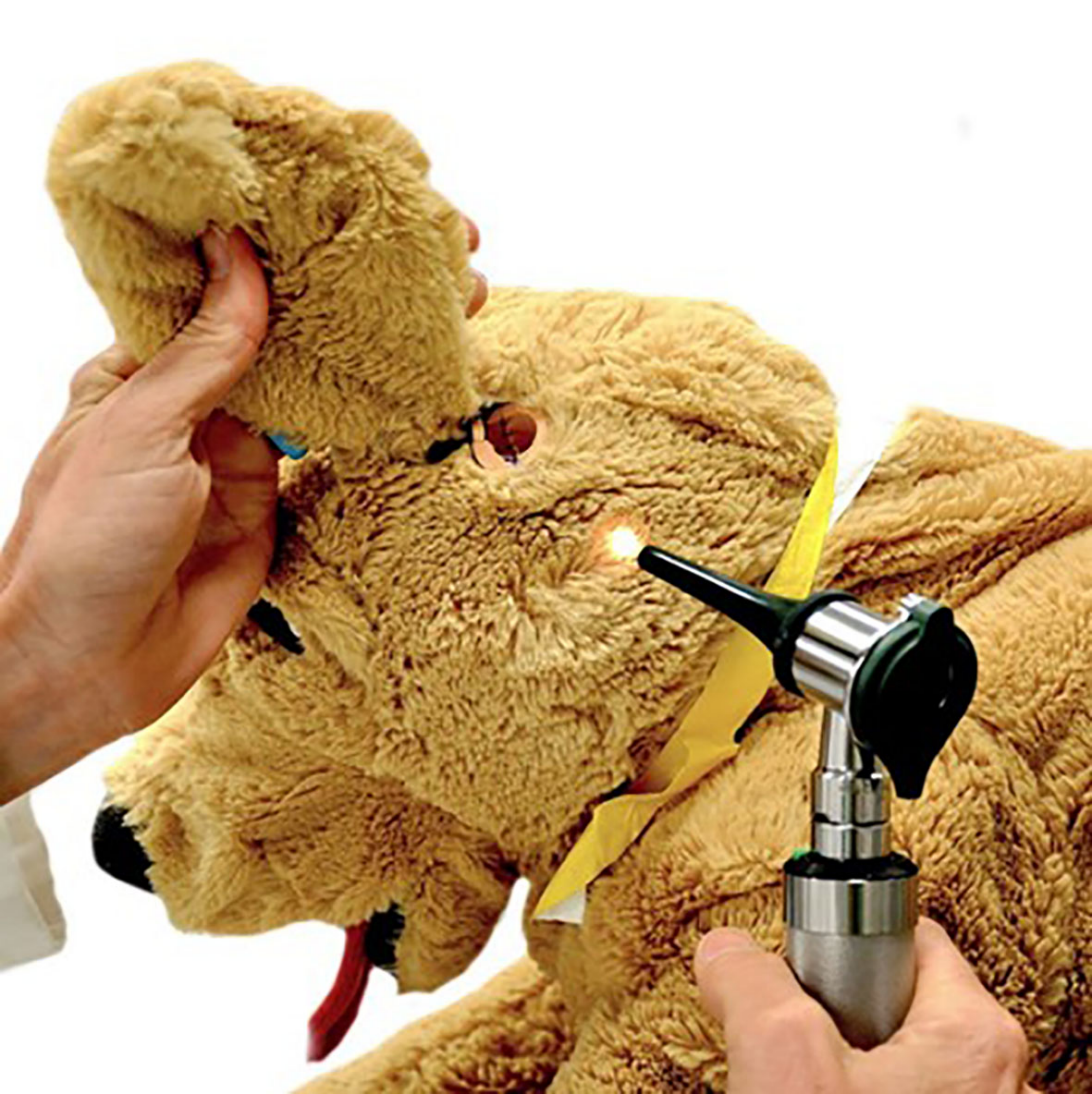
The canine otoscopy model, created at LMU-CVM

**Figure 7.**
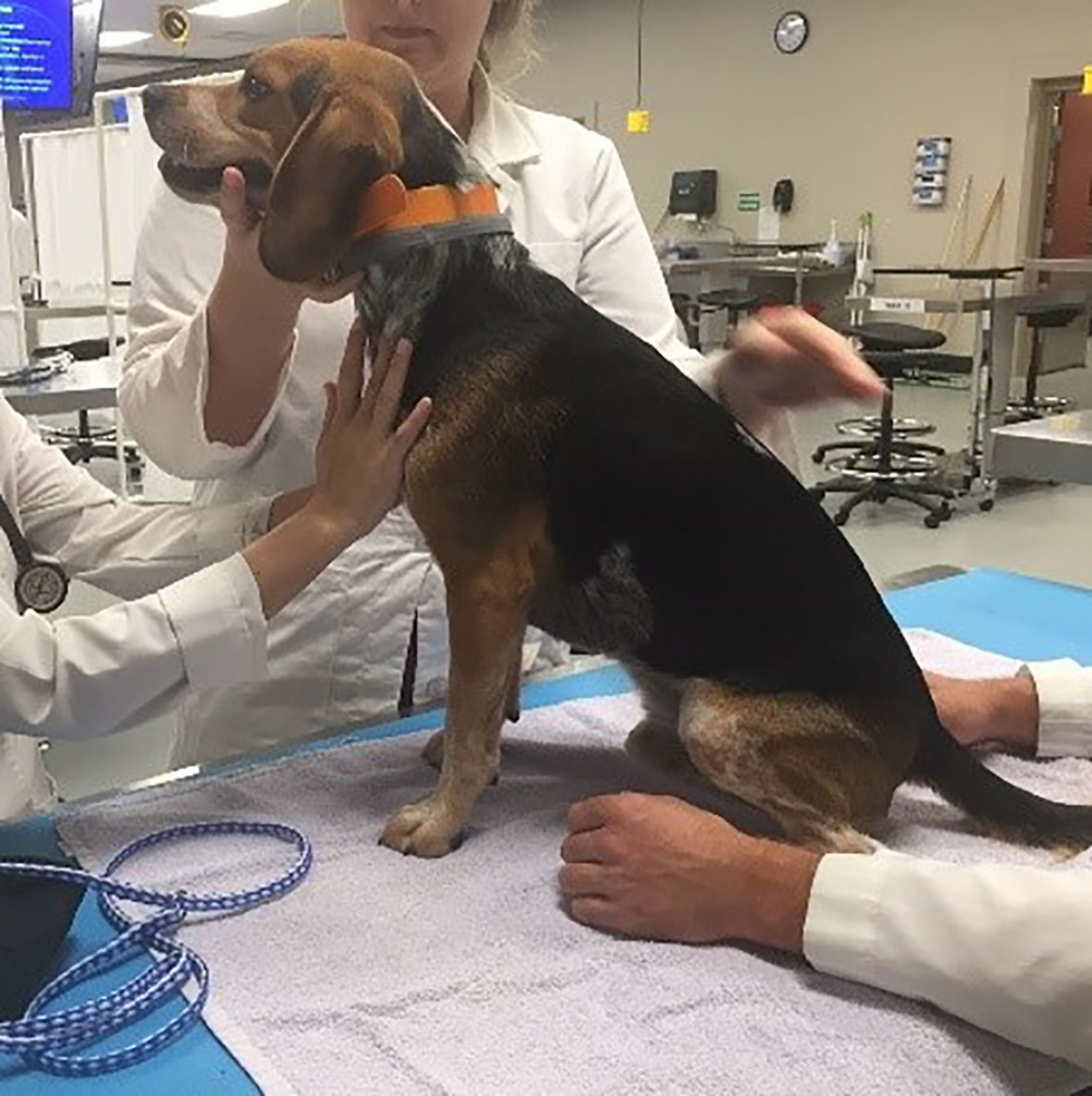
A student palpates the jugular vein in preparation for mock venipuncture

**Figure 8.**
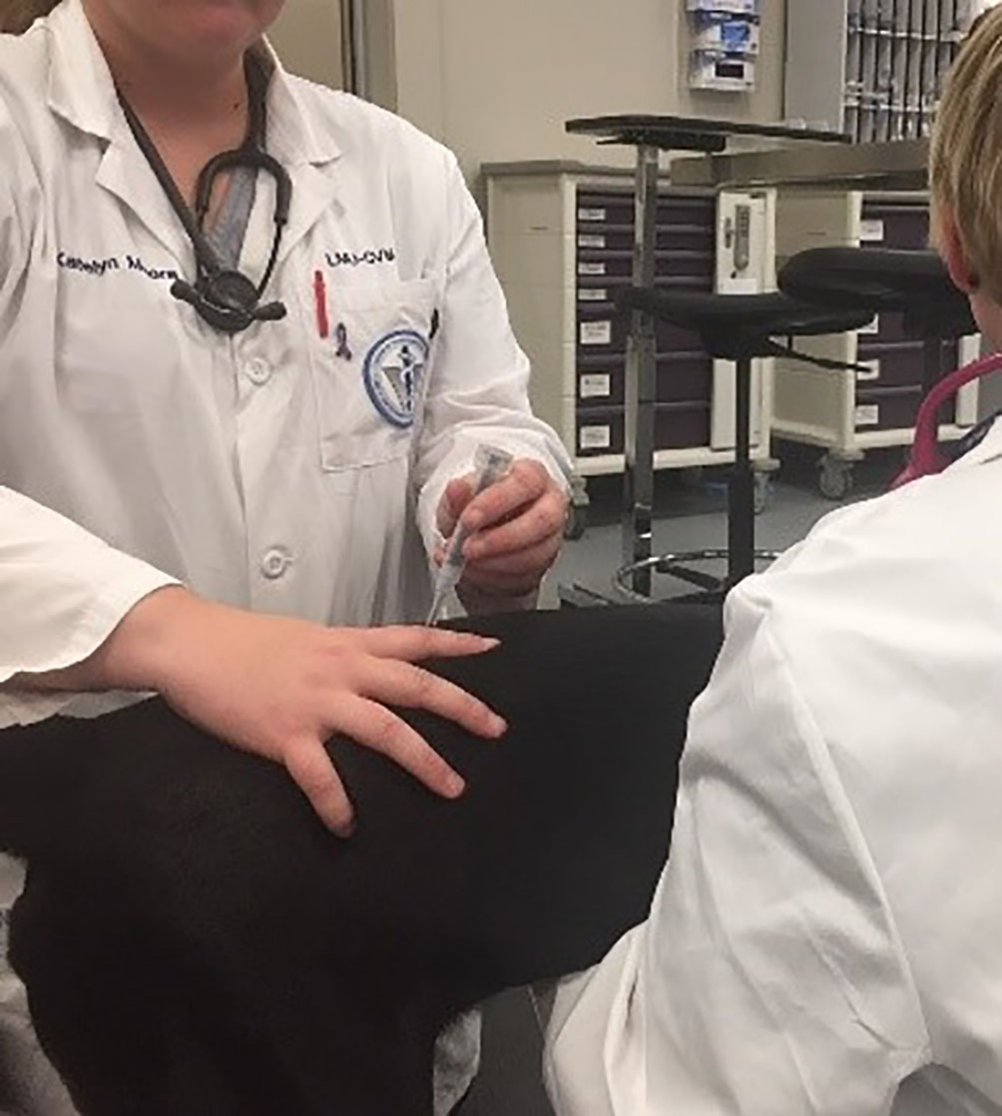
A student performs mock intramuscular injection in preparation for administration of anesthetic premedication

**Figure 9.**
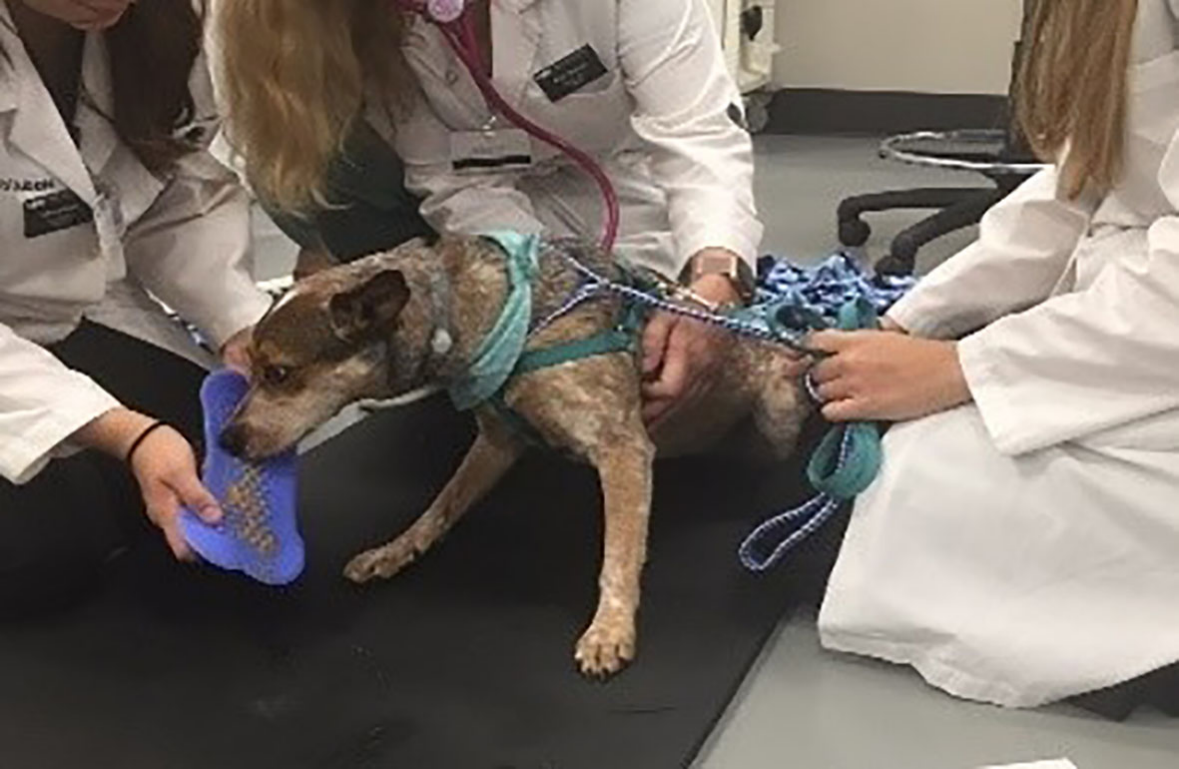
Food is offered to distract a patient during venipuncture

**Figure 10.**
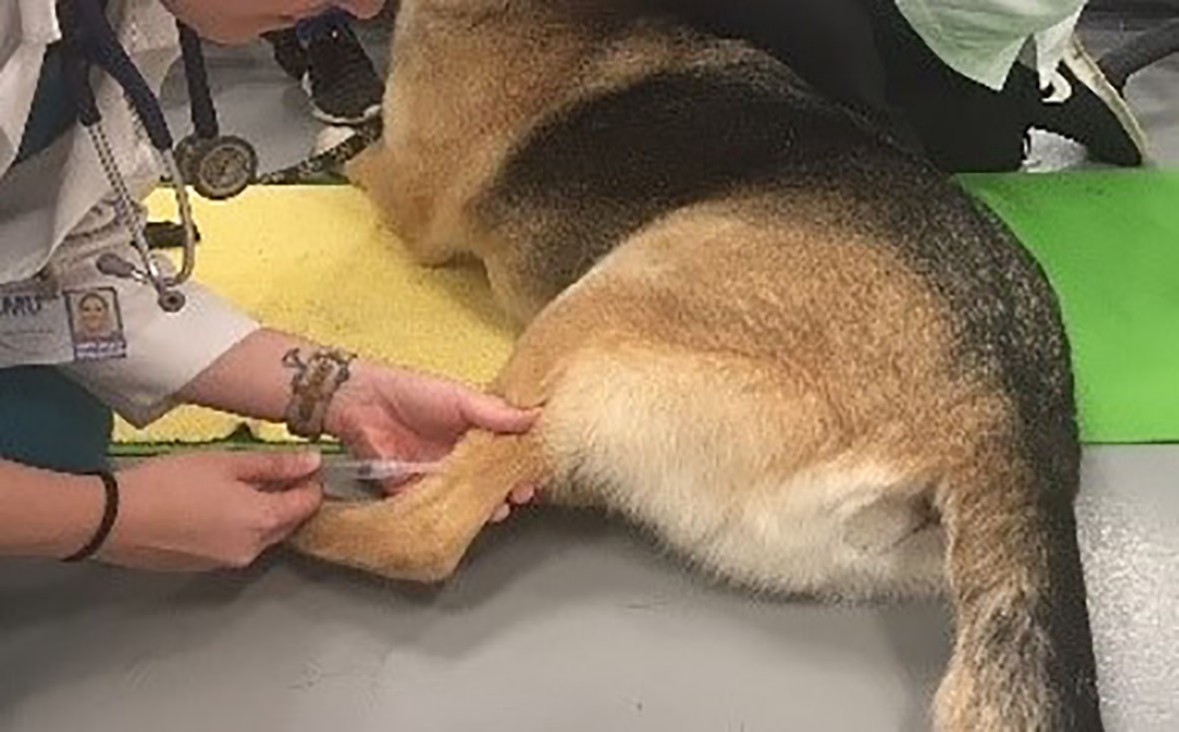
A student performs lateral saphenous venipuncture, which is less stressful for some dogs

**Figure 11.**
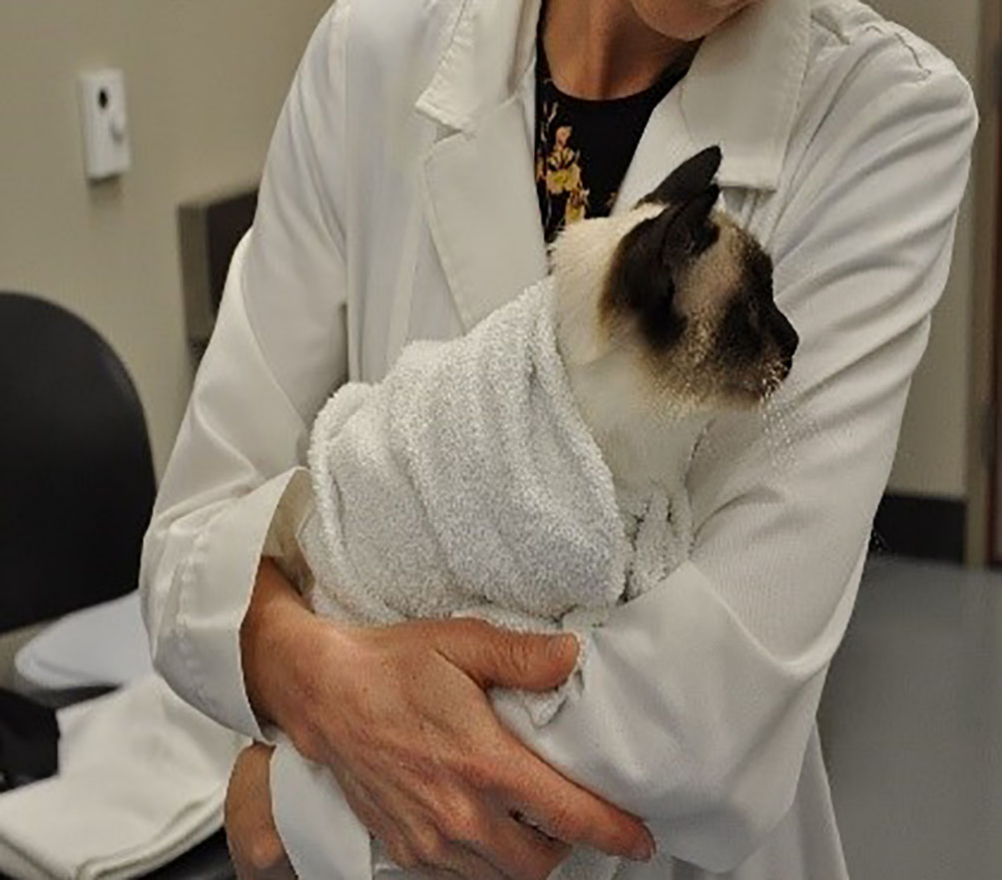
A student towel wraps a cat for a calming moment

**Figure 12.**
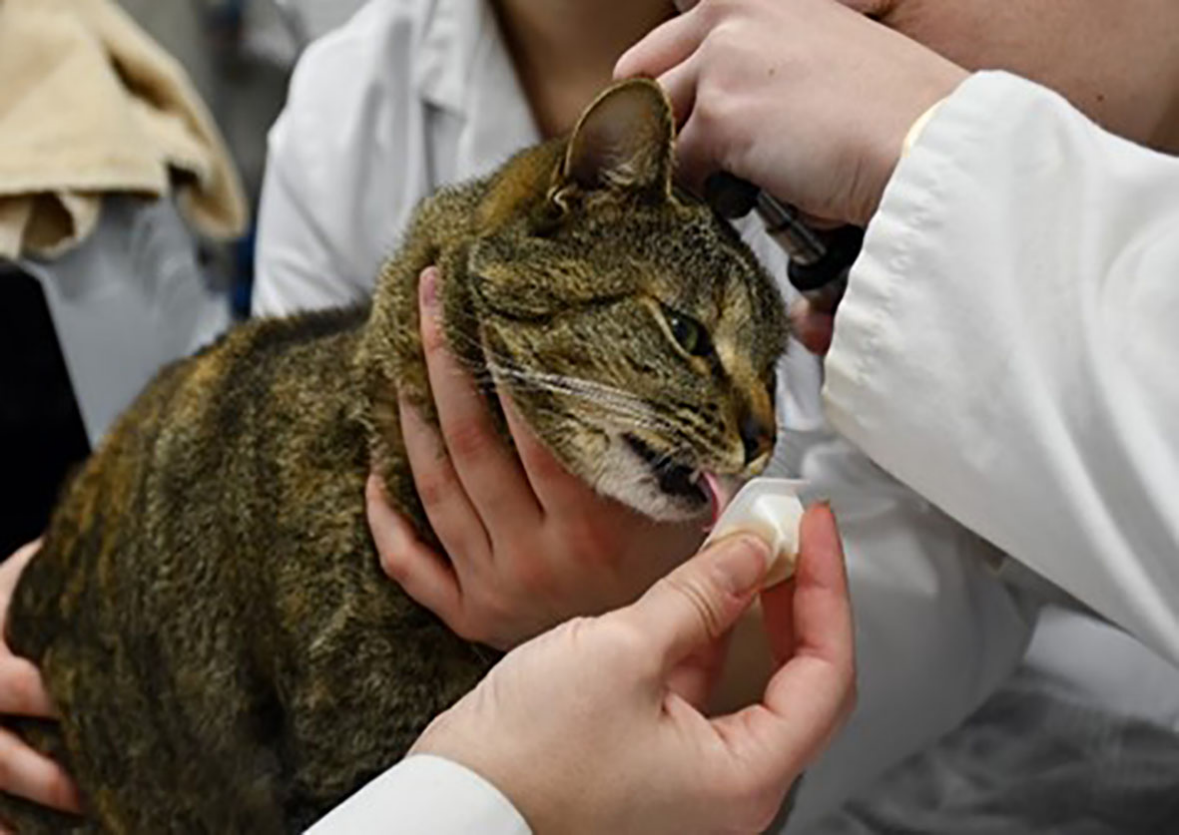
Food is offered to a cat during an examination

**Figure 13.**
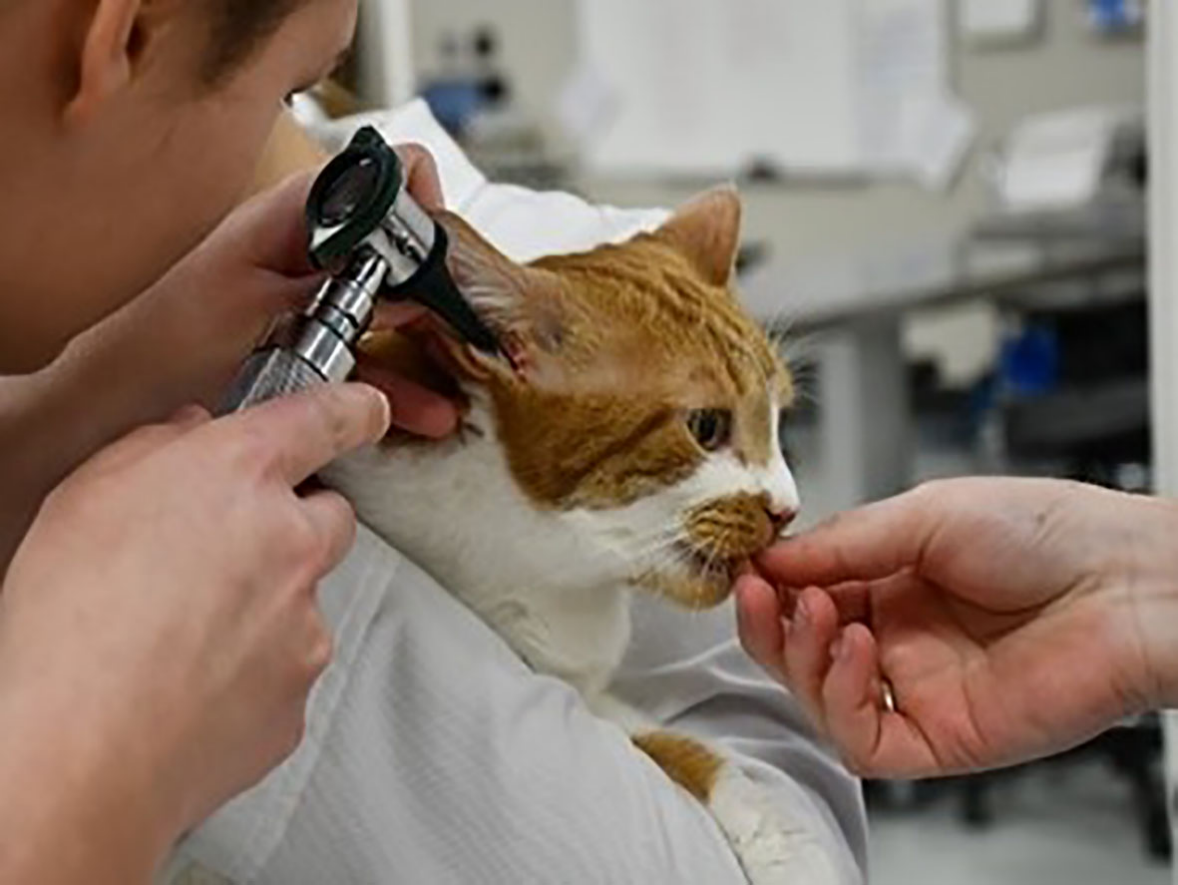
Treats are offered to distract a cat during otoscopic examination

## Appendices

### Appendix 1 Lesson Plans for Gentle Animal Handling Laboratories

#### Semester 1 Canine Handling Laboratory

*Duration:* 2 hours

*Instructor to Student Ratio* 1:10

*Lab Objective:* The goals of this clinical skills laboratory are to initiate a discussion of canine body language, review the fear, anxiety, and stress (FAS) scale; to learn the common canine venipuncture sites; to practice positioning of a model and then a live dog for examination or mock venipuncture; and to practice walking a dog onto a scale. Students are to perform these tasks consistent with gentle handling principles.

*Lab Overview:* Prior to the laboratory session, students complete the Fear Free certification course and review a PowerPoint or iBook that explains canine body language and diagrams canine venipuncture sites. Students are taught to examine a patient’s tail, ears, eyes, mouth and posture or (TEMP) as an introduction to body language.

In this laboratory session, students practice recognizing canine body language based on a series of photographs. Students then practice placing their model patient in sitting, sternal or standing restraint in small groups, adjusting their handling on the table or the floor based on the size of the model patient. Students practice handling stuffed dog models for mock venipuncture using the cephalic vein, jugular vein or lateral saphenous vein. ( Figure 1) Students discuss how to adjust the environment to ensure their patient will experience a decreased amount of FAS. The students are asked to take into consideration what the dog may see, hear, feel, smell or taste while in that laboratory. Environmental adjustments may include placing separation curtains between dogs, using quiet voices, using pheromone diffusers, judicious use of treats, slow and considerate touch or moving to a private area.

Once a student has practiced on the dog models, the student will go to the kennel to assess canine body language and assign a FAS score to their live dog. The student then develops a handling plan ( Herron and Shreyer, 2014) and gathers any needed materials, such as mats and a variety of treats. The student brings the dog into the laboratory or into a private room. Pheromone diffusers are in place in the laboratory. Students work in groups of three or four and take turns practicing handling. The laboratory goal is not getting every dog into every position to practice, but rather determining the best handling method for the patient to complete the procedure, which is positioning for mock venipuncture. ( Figure 2) Students practice weighing the dogs on a scale in a gentle manner by walking along the side of the dog, avoiding standing in front of the dog and using treats as a lure. Students consider needs versus wants and make adjustments to handling. Students are taught that if a patient struggles for more than 3 seconds then the handling technique should be adjusted. If a patient struggles for more than 3 times, the patient gets a break while a new handling plan is developed.

Equipment and supplies:

• Yoga mats, blankets and towels
• Clip leashes with double handles
• Slip leashes
• Harnesses
• Small food bowls
• Wearable treat pouches, durable plastic spoons, Kong ^©^ Classic toys of various sizes
• Melissa & Doug stuffed animals (Rottweiler, Black Lab, Yellow Lab. Chihuahua, Jack Russell Terrier, and Basset Hound)
• Variety of high value treats: dry kibble, peanut butter, squeeze cheese, soft and hard treats, training treats, Kong paste, pretzel sticks and dental chews
• Treat mats with suction cups
• Water bowls
• Towels
• Curtains to separate canines
• Pheromone spray
• 2 scales
• Canine body language form
• FAS scale

*A* *ssessment:* The student is assigned one of the practiced skills, then must demonstrate that skill on a canine model. Skills assessment examples include: describe at least 4 body language signs in your dog and assign your dog a FAS score. Skills from this and any other laboratory session may also be tested on students’ OSCE at the end of their semester.

*Variations:* Head halter style leaders or front clip harnesses may be worn by dogs that have been trained to wear them.

#### Semester 1 Feline Handling Laboratory

*Duration:* 1.5 hours

*Instructor to Student Ratio* 1:10

*Laboratory Objective*: The goal of this clinical skills laboratory is to initiate discussion of feline body language; to practice positioning of model cats; to practice multiple feline towel wrap methods ( Yin, 2009); to practice methods of removing (or not) cats from carriers ( Anseeuw *et al.,* 2006; Rodan *et al.,* 2011); to discuss transportation of cats within a hospital; and to learn the common feline venipuncture sites used in veterinary medicine.

*Laboratory Overview:* Prior to the laboratory session, students review a PowerPoint or iBook that demonstrates feline body language and diagrams feline venipuncture sites. Students are taught to examine a patient’s tail, ears, eyes, mouth (whiskers) and posture or TEMP as an introduction to body language.

In this laboratory session, students practice recognizing feline body language based on a series of photographs. Students discuss how to adjust the environment to ensure their patient experiences a decreased amount of FAS. The student should take into consideration what a cat may see, hear, feel, smell or taste while in that laboratory. This laboratory covers removing cats from carriers (or not) and the pros and cons of some of the available types of cat carriers and the importance of training a cat to enter a carrier. Students then work in groups of 3 or 4 to practice handling cat models for mock venipuncture from the cephalic vein, jugular vein or medial saphenous vein. Students practice sitting, sternal, standing and lateral restraint methods. Students then practice multiple methods of towel wrapping cats and discuss the best times to use towel wraps ( Yin, 2009). Students demonstrate placing cat models in cat bags and place cone style masks on the models. Students practice methods to capture escaped cats including using towels or a mesh EZ nabber plus towels. Students put on leather gloves to experience the lack of dexterity and the lack of feedback on how tightly you may be holding a patient when wearing the gloves. This laboratory works only with model cats; live cats are introduced later in the curriculum.

Equipment and supplies:

• Feline models - Folkmanis rag doll cat puppet or other stuffed cat
• Front and top load carriers
• Towels
• Cat gloves
• Cat bag
• Cat muzzles - nylon and cone style
• Mesh EZ nabber
• Feline body language form
• FAS scale

*Assessment:* The student is assigned one of the practiced skills, then must demonstrate that skill on a feline model. Skills assessment examples include: wrap the cat in a towel and demonstrate a gentle handling method for medial saphenous venipuncture.

#### Semester 1 Introduction to Canine Physical Examination

*Duration:* 2 hours

*Instructor to Student Ratio* 1:10

*Laboratory Objective:* The goal of this laboratory is to practice gentle handling of a live dog while learning to perform a physical examination.

*Laboratory Overview:* Prior to the laboratory session, students review a PowerPoint or iBook which includes video recordings of new skills.

In this laboratory sessions, students work in groups of 3 or 4 and take turns performing a physical examination on a dog. Students may review handling techniques using models prior to handling the live dog. One student handles the patient based on skills learned in the previous canine handling laboratory; one student performs the examination; and one to two other students observe, keep notes on physical examination findings, and may offer feedback. Students must determine how to best handle the patient in a gentle manner while still completing the physical examination. Students must consider needs versus wants and make adjustments to handling. Students are taught that if a patient struggles for more than 3 seconds then the handling technique should be adjusted. If a patient struggles for more than 3 times, the patient gets a break.

Equipment and Supplies:

• Yoga mats and towels
• Clip leashes with double handles
• Slip leashes
• Wearable treat pouches, durable plastic spoons, Kong© Classic toys of various sizes
• Melissa & Doug stuffed animals (Rottweiler, Black Lab, Yellow Lab. Chihuahua, Jack Russell Terrier, Pug and Basset Hound)
• Variety of high value treats including: dry kibble, peanut butter, squeeze cheese, soft and hard treats, training treats, kong paste, pretzel sticks and dental chews
• Water bowls
• Curtains to separate canines
• Physical examination form
• Canine body language form
• FAS scale

*Assessment:* Each student is assigned one of the practiced skills, then must demonstrate that skill on the model or live canine. Skills assessment examples include: palpate and report your dog’s femoral pulse rate.

*Variations:* Time permitting, students may rotate to another group and perform an examination on an additional 2 to 3 dogs.

#### Semester 2 Canine Physical Examination - Focus on Ophthalmology and Oral Cavity

*Duration:* 2 hours

*Instructor to Student Ratio* 1:6

*Laboratory objective* *:* The goals of this laboratory are to complete a basic ophthalmic examination and an oral examination on a live dog utilizing gentle handling techniques. Students also practice assembling ophthalmoscopes and otoscopes.

*Laboratory overview* *:* Prior to this laboratory session, students read a brief article about a basic ophthalmic examination and view video demonstrations of oral and ophthalmic examinations.

In this laboratory session, students use the handling and physical examination skills learned in previous laboratories. The students should also attempt to examine the structures of the eye other than the fundus (lids, lashes, nictitating membrane, conjunctiva, sclera, anterior chamber, iris, pupil, and note any ocular discharge or change in symmetry) and the face, lips, teeth, gums, vestibule, tongue, hard palate and oropharynx as part of the oral examination. This laboratory requires students to recognize how FAS changes with examination procedures and to then to make adjustments to lower the level of FAS. Students must decide needs versus wants.

Equipment and Supplies:

• Canine handling supplies
• Tongue depressors
• Ophthalmoscope sets
• Direct lens
• Transilluminator

*Assessment:* The student is assigned one of the practiced skills, then must demonstrate that skill on a dog or a model. Skills assessment examples include: assemble an ophthalmoscope then examine and describe your dog’s sclera or examine your dog’s teeth and describe any abnormalities.

#### Semester 2 Canine Venipuncture Laboratory

*Duration:* 2 hours

*Instructor to Student Ratio* 1:6

*Laboratory objectives:* The goal of this laboratory is to practice venipuncture techniques on a live dog while utilizing gentle handling.

*Laboratory overview:* Prior to the laboratory session, students review a PowerPoint or iBook which demonstrates venipuncture sites and techniques.

In this laboratory session, students practice venipuncture on a canine forelimb model ( Figure 3) and a feline medial saphenous vein model ( Figure 4), emphasizing gentle handling on the models. Students work in pairs. Once an instructor has approved of both students’ technique, the students are assigned a live dog that has been pre-medicated with trazadone 2-4 mg/kg by mouth 60-90 minutes prior. Each pair of students will assess their dog’s body language and develop a handling plan. Each student can attempt venipuncture a limited number of times and locations on a live dog but must have an instructor present for coaching during attempted venipuncture. While waiting for an instructor to supervise venipuncture, students perform a physical examination. One student is responsible for monitoring FAS changes during handling and making any needed adjustments while the other student performs the physical examination, then the students trade roles.

Equipment and supplies:

• Canine cephalic venipuncture models
• Feline medial saphenous venipuncture models
• Canine handling supplies
• Venipuncture supplies
  • 22G needles
  • 20 G needles
  • Red tp tubes
  • Purple top tubes
  • 1 cc syringes
  • 3 cc syringes
  • 25 G butterfly catheters
• Treat variety
• Trazadone
• Sedation with acepromazine and hydromorphone as deemed necessary ( Herron and Shreyer, 2014)

*Assessment:* Each student must demonstrate to an instructor a competent venipuncture technique on a model prior to being allowed to attempt venipuncture on a live dog.

#### Semester 3 Canine Physical Examination - Focus on Ophthalmology and Otoscopy

*Duration:* 3 hours

*Instructor to Student Ratio* 1:10

*Laboratory objective:* The goals of this laboratory are to review the physical examination; to introduce students to otic anatomy; to review the ophthalmic examination; and to practice a brief direct and indirect fundic examination on a live dog while utilizing gentle handling techniques. This laboratory uses a canine fundic and ophthalmic model as well as live dogs. Students will rotate through a video otoscope station.

*Laboratory overview:* Prior to the laboratory session, students read a brief article and review videos demonstrating a direct and an indirect fundic examination on a model.

In this laboratory session, students will perform direct and indirect ophthalmoscopy utilizing models first ( Figure 5). Students may then use a live dog to repeat the physical examination then attempt direct and indirect ophthalmoscopy. Students practice otoscopy on a canine model. ( Figure 6) Students rotate through a station that demonstrates a video otoscope on a canine model. Using the skills learned in previous clinical skills laboratories, students should recognize how FAS changes with procedures and then to make adjustments to lower the level of FAS. These adjustments may include changing the location the patient is examined, deciding needs versus wants, adding curtains, giving the patient a break or deciding that the patient needs medication prior to further examination.

Equipment and supplies:

• Canine handling supplies
• Canine ophthalmology supplies
• Canine eye models
• Canine ear models
• Video otoscope set up with a dog model

*Assessment:* The student is assigned one of the practiced skills, then must demonstrate that skill on a model. Skills assessment example: demonstrate direct ophthalmoscopy on a model or demonstrate otoscopy on a model.

#### Semester 3 Canine Clicker Training Laboratory

*Duration:* 1.5 hours

*Instructor to Student Ratio* 1:8

*Laboratory objectives* *:* The goal of this laboratory is to introduce students to clicker training, positive reinforcement and to initiate training basic commands with a secondary goal of increasing the adoption potential of the shelter dogs included in the laboratory.

*Laboratory overview:* This laboratory takes place in a large open room with curtains to separate dogs as needed. Several private rooms are available as well as a semi-private hallway. When possible, a guest behaviorist leads this laboratory. Students are introduced to positive reinforcement and training through a PowerPoint presentation. Students then work in pairs and go through several exercises to gain familiarity with clicker training. First, students click and place treats in a cup and return the treat hand to a neutral position, then practice with a timer. Finally, students practice placing a treat in a cup each time a dog sits during a video. Once students have gone through the practice exercises, they retrieve the dogs from the kennel and complete a brief exercise on leash walking a dog while in the hospital ( Yin, 2009) and walk the dogs across a parking lot to the laboratory. Students practice marking wanted behaviors with the clicker, delivering treats quickly for desired behaviors, and returning the treat hand to a neutral position. Each dog is trained for up to twenty minutes. Some dogs learn to sit, down, high five and even roll over. For some dogs, this session becomes a desensitization period and they receive treats and petting for being in the lab. Students must recognize FAS and take steps to reduce any FAS.

Equipment and supplies:

• Supplies listed under canine handling, except for models
• Curtains
• Clickers
• Treat pouches
• Targets

*Assessment:* There is no formal assessment for this laboratory. Students are encouraged to continue clicker training during scheduled daily enrichment sessions with the dogs.

#### Semester 4 Communication Skills and the Physical Examination

*Duration:* 1 hour

*Instructor to Student Ratio* 1: 12

*Laboratory Objectives:* The goal of this laboratory is for the veterinary student to communicate that they are performing a physical examination and to explain the examination findings to a simulated client. This laboratory uses dogs owned by faculty and staff. Students are required to use gentle handling techniques.

*Laboratory overview:* Students work in groups of 4 and rotate through various scenarios. Students take turns playing the role of client, pet handler, veterinarian and observer ( Jackson and Back, 2011). Students practice gentle handling as part of this laboratory.

Equipment and Supplies:

• Canine handling supplies minus models
• Faculty and staff owned pets
• Communication feedback forms

*Assessment:* Students complete online feedback forms based on communication skills training.

#### Semester 5 Canine Gentle Handling Laboratory

*Duration:* 1.5 hours

*Instructor to Student Ratio* 1:8

*Laboratory objective:* The goal of this lab is to practice gentle handling while performing a physical examination, mock venipuncture, and anesthesia premedication of a live dog prior to beginning surgery laboratories.

*Laboratory overview:* This laboratory is a mock surgery work up day and requires the students to perform each step of surgery work up and anesthesia premedication. Students work in groups of three. Students must observe and interpret the dog’s body language, assign a FAS score, then gather any necessary supplies and adjust the environment prior to removing the dog from the kennel. Students then retrieve the dogs from the kennels, perform a physical examination and position the dogs for mock venipuncture ( Figure 7) and administration of anesthesia premedication by intramuscular injection ( Figure 8). Students must continuously monitor for any FAS and take steps to reduce FAS.

Equipment and supplies:

• Canine handling supplies minus the models
• Venipuncture supplies

*Assessment:* Students complete an online summary of their proposed handling plan for surgery work up day as the assessment.

#### Semester 5 Surgery Work up Laboratory

*Duration:* 2 hours

*Instructor to Student Ratio* 1:9

*Lab Objective:* Under the supervision of a veterinarian, students complete a physical examination, perform venipuncture, and analyze blood work and necessary diagnostics prior to performing a spay or neuter surgery on local shelter dogs and cats.

*Lab Overview:* Cats and dogs are housed and examined in separate rooms by species. Students are directed to read the patient’s body language, develop a handling plan and gather any needed supplies. The handling plan should include any adjustments to the environment including dimming lights, offering a variety of treats ( Figure 9), using pheromones and separating patients into private rooms as needed. Students perform a complete physical examination and are allowed 2 attempts at venipuncture at which point a veterinarian or veterinary technician will complete venipuncture if needed. ( Figure 10) Due to the potentially fractious nature of cats, students must have a veterinarian or veterinary technician present to attempt any feline venipuncture. Chlorhexidene solution rather than alcohol is used for feline venipuncture ( Carney *et al.,* 2012). Any behavioral information should be recorded in the patient’s medical record. If a dog struggles for more than 3 seconds or for more than 3 attempts a new handling technique is used or the patient is medicated or sedated. If a cat struggles for more than 2 seconds or for more than 2 attempts a new handling technique is used or the patient is medicated. ( Figure 11)

On surgery day, students must continue handling their patients in a gentle manner, although due to preanesthetic fasting requirements, the use of treats is curtailed. Students may utilize chicken broth ice cubes if needed to distract during injections.

*Assessment:* This laboratory has a series of rubrics assessing patient workup and medical records.

#### Semester 6 Feline Gentle Handling Laboratory

*Duration:* 1.5 hours

*Instructor to Student Ratio* 1:6

*Lab objective:* The goal of this laboratory is for students to gain experience handling live cats in a gentle manner.

*Lab Overview:* Faculty, staff, and students graciously volunteer their tolerant cats for this laboratory. With the owners’ permission, the cats are given gabapentin 50-100 mg by mouth the night before and the morning of the laboratory. Breakfast is withheld the day of the laboratory to improve the cats’ response to treats.

Students work in groups of 3 or 4. While the cats are still in their kennels, students must assign a FAS score and verbally describe their cat’s body language to an instructor. Students then develop a handling plan and a plan B. Students then gather any needed supplies, adjust the environment and help decide if the cat needs a private room. Only after these steps are complete can the students handle the cats. Students perform a physical examination, place the cat into positions for mock venipuncture and may practice towel wrapping cats who are tolerant. Treats are used to distract and encourage. ( Figures 12 and 13)

Actual feline handling time is approximately 20 minutes. If a cat struggles for more than 2 seconds or for more than 2 attempts a new handling technique is used or the patient is given a handling break.

Equipment and supplies:

• Non slip mats
• Blankets
• Towels pre-sprayed with Feliway
• Carriers with front and top load
• Variety of tempting treats (marshmallows, whipped cream, butter, tuna, salmon, soft and crunchy treats)
• bowls/plates
• Large spoons
• Feliway spray and diffuser
• Catnip spray
• Catnip leaves
• Toys
• Canned cat food
• Curtains
• Environment adjusted based on patient preferences including lights, noise, smells, touch

*Assessment:* Students receive a global score based on their description of body language, determination of a FAS score, and group participation.

**Semester 6 Canine Physical Examination** - Focus on ophthalmology with fundic exam and otoscopy

*Duration:* 2 hours

*Instructor to Student Ratio* 1:6

*Lab Objective:* The goal of this laboratory is to complete a fundic examination on a dog and to perform an otoscopic examination.

*Lab overview:* Students work in pairs to perform a physical examination. Students use the fundic eye models and canine ear models to review the ophthalmic examination and the otoscopic examination respectively. Dogs receive 1 drop of tropicamide in each eye. The laboratory lights are dimmed and remain dimmed throughout the laboratory session. Students attempt the fundic examination on a non-sedated dog. Small amounts of squeeze cheese may be used to facilitate a positive experience for the dogs. To facilitate an easier learning experience and a less stressful experience for the dogs, dogs are then sedated with acepromazine 0.03 mg/kg and hydromorphone 0.05 mg/kg intravenously. Students must calculate each dog’s dosage and administer the sedation intravenously using the previously learned gentle handling techniques. Students may attempt otoscopy for dogs that are cooperative and non-painful. For some dogs, otoscopy is postponed until dogs are anesthetized prior to surgery later in the semester. Dogs are returned to kennels that have dimmed lighting.

Equipment and supplies:

• Canine handling supplies
• Canine ophthalmology supplies
• Canine fundic eye models
• Canine ear models
• Acepromazine
• Hydromorphine
• 1 ml syringes and needles
• Tropicamide

*Assessment:* Each student must demonstrate the proper technique for a direct or an indirect fundic examination on a dog model.

**Semester 6 Surgery Work up Laboratory:** See description under Semester 5. This laboratory is repeated another three times in semester 6.

## Declarations

Julie Hunt is an Associate Editor of MedEdPublish.
