# Supplementary material for: Teaching Gentle Canine and Feline Handling as a Veterinary Clinical Skill
Source: MedEdPublish (2016). 2021 Jun 7;10:158. [Version 1] doi: 10.15694/mep.2021.000158.1 (PMC10939569; doi:10.15694/mep.2021.000158.1)
Supplement: Peer review history

# Reviewer report: Reviewer response for version 1

**DOI:** 10.21956/mep.18978.r27188

**Published:** 2021-10-11

**Authors:** Ken Masters

**Affiliations:** 1 Sultan Qaboos University

This review has been migrated. The reviewer awarded 4 stars out of 5 A really interesting paper on teaching gentle canine and feline handling skills.While the body of the paper is a little short, the Appendix gives a great deal of detail on the lessons with useful text and photographic illustrations (with a variety of animals and animal models). As the paper is on a new educational tool, the focus is on supplying the reader with detailed information of the tool, including estimated time and equipment required, in order to implement; the paper has performed this task very well. In addition, the authors have responsibly indicated the challenges, so as to ensure that readers do not simply rush ahead with implementation, and should consider the difficulties ahead so that the program can be properly implemented.Overall, a useful paper. I look forward to further, more detailed research papers on the topic.

Reviewer Expertise:

NA

No decision status is available

# Reviewer report: Reviewer response for version 1

**DOI:** 10.21956/mep.18978.r27187

**Published:** 2021-07-14

**Authors:** Richard Hays

**Affiliations:** 1 James Cook University

This review has been migrated. The reviewer awarded 4 stars out of 5 This is a well-written, thoughtful article that describes clearly the approach to teaching clinical skills to veterinary students in one vet school, but based on more widely adopted principles. I am an educator in medical programs but recognised that the program is based on sound principles and practice that can be found in some medical programs, where the focus is similarly on skills that are both better accepted by patients and can make a difference to the quality of care. An analogy (not entirely equivalent) that comes to mind is how doctors approach small children makes a big difference to achieving appropriate history and examination findings. This represents a substantial investment in resources but should make a difference to clinical care. To date there have been some in-house evaluations that have led to refinements in the program, but longer term evaluations through to beyond graduation are necessary to determine the long term value. I look forward to reading those evaluations in the future.

Reviewer Expertise:

NA

No decision status is available
